# *catena*-Poly[[diaqua­strontium(II)]-bis­[μ-2-(3-benzoyl­phen­yl)propano­ato]]

**DOI:** 10.1107/S160053681003429X

**Published:** 2010-09-04

**Authors:** Zhu-Yan Zhang, Nan Yu, Min Li, Ning-Xin Wu, Bing-Yi Liu

**Affiliations:** a School of Pharmaceutical Science, Harbin Medical University, Harbin 150081, People’s Republic of China

## Abstract

In the title coordination polymer, [Sr(C_16_H_13_O_3_)_2_(H_2_O)_2_]_*n*_, the Sr^II^ cation is eight-coordinated by six O atoms from four different 2-(3-benzoyl­phen­yl)propano­ate ligands and two O atoms of two water mol­ecules in a distorted dodeca­hedral geometry. Adjacent Sr^II^ cations are bridged by two 2-(3-benzoyl­phen­yl)propano­ate ligands, forming an infinite chain along the *b* axis; the chains are further linked by inter­molecular O—H—O hydrogen bonds into a three-dimensional supra­molecular network.

## Related literature

For the crystal structures of metal complexes of the 2-(3-benzoyl­phen­yl)propano­ate anion, see: Tahir *et al.* (1997 ▶); Zhang *et al.* (2007*a* ▶,*b* ▶). 
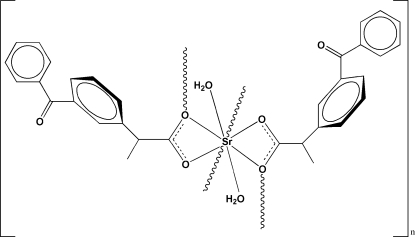


## Experimental

### 

#### Crystal data

[Sr(C_16_H_13_O_3_)_2_(H_2_O)_2_]
*M* *_r_* = 630.18
Monoclinic, 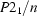

*a* = 18.665 (4) Å
*b* = 8.0406 (16) Å
*c* = 19.377 (4) Å
β = 93.26 (3)°
*V* = 2903.4 (10) Å^3^
*Z* = 4
Mo *K*α radiation
μ = 1.91 mm^−1^
*T* = 295 K
0.30 × 0.25 × 0.18 mm

#### Data collection

Rigaku R-AXIS RAPID diffractometer
Absorption correction: multi-scan (*ABSCOR*; Higashi, 1995 ▶) *T*_min_ = 0.598, *T*_max_ = 0.725
22141 measured reflections
5107 independent reflections
2941 reflections with *I* > 2σ(*I*)
*R*_int_ = 0.087

#### Refinement

*R*[*F* ^2^ > 2σ(*F* ^2^)] = 0.050
*wR*(*F* ^2^) = 0.136
*S* = 1.08
5107 reflections
385 parameters
7 restraints
H atoms treated by a mixture of independent and constrained refinement
Δρ_max_ = 0.69 e Å^−3^
Δρ_min_ = −0.96 e Å^−3^

### 

Data collection: *RAPID-AUTO* (Rigaku, 1998 ▶); cell refinement: *RAPID-AUTO*; data reduction: *CrystalStructure* (Rigaku/MSC, 2002 ▶); program(s) used to solve structure: *SHELXS97* (Sheldrick, 2008 ▶); program(s) used to refine structure: *SHELXL97* (Sheldrick, 2008 ▶); molecular graphics: *ORTEPII* (Johnson, 1976 ▶); software used to prepare material for publication: *SHELXL97*.

## Supplementary Material

Crystal structure: contains datablocks I, global. DOI: 10.1107/S160053681003429X/ng5018sup1.cif

Structure factors: contains datablocks I. DOI: 10.1107/S160053681003429X/ng5018Isup2.hkl

Additional supplementary materials:  crystallographic information; 3D view; checkCIF report

## Figures and Tables

**Table 1:** Selected bond lengths (Å)

Sr1—O5^i^	2.496 (3)
Sr1—O1^ii^	2.514 (3)
Sr1—O1*W*	2.529 (4)
Sr1—O2	2.557 (4)
Sr1—O2*W*	2.559 (4)
Sr1—O4	2.590 (4)
Sr1—O5	2.772 (3)
Sr1—O1	2.816 (3)

Symmetry codes: (i) 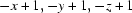; (ii) 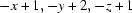.

**Table 2:** Hydrogen-bond geometry (Å, °)

*D*—H⋯*A*	*D*—H	H⋯*A*	*D*⋯*A*	*D*—H⋯*A*
O1*W*—H1*W*2⋯O2^i^	0.85 (1)	1.83 (1)	2.678 (5)	171 (6)
O2*W*—H2*W*2⋯O4^ii^	0.85 (1)	1.89 (2)	2.724 (5)	166 (6)

Symmetry codes: (i) 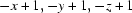; (ii) 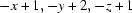.

**Fig. 1.**
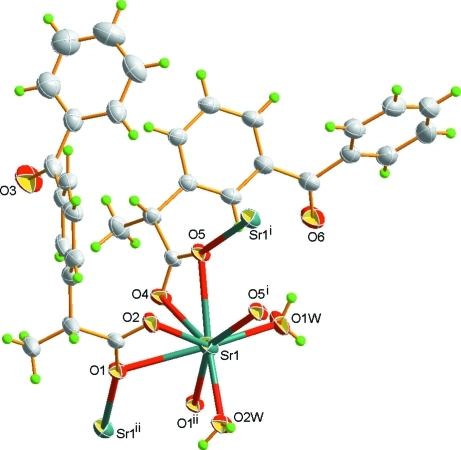
Molecular structure of the title polymer with 30% probability ellipsoid for the non-H atoms.

**Fig. 2.**
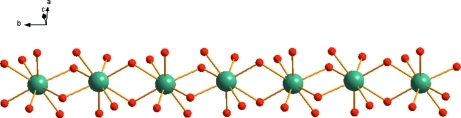
1-D chain structure of the title polymer (the C atoms are omitted for clarity).

## supplementary crystallographic information

### Comment

2-(3-Benzoylphenyl)propanoic acid is known as nonsteroidal anti-inflamatory drug. To date, some reports on the structure of its metal complexes (Tahir *et al.*, 1997; Zhang *et al.*, 2007a,b) have been documented. In this paper, we present a novel 1-D Sr^II^ coordination polymer, [Sr(C_16_H_13_O_3_)_2_(H_2_O)_2_]_n_, (I). Its crystal structure is described here. As illustrated in Fig. 1, the complex (I) consists of one Sr^II^ cation, two 2-(3-benzoylphenyl)propanoate ligands and two coordinated water molecules. The local coordination environment around the Sr^II^ cation can be described as a distort dodecahedron, involving six oxygen atoms of four different 2-(3-benzoylphenyl)propanoate ligands and two coordinated water molecules. The 2-(3-benzoylphenyl)propanoate acts as bidentate bridging ligand to link adjacent Sr^II^ cations, leading to a 1-D chain along *b* axis direction (Fig. 2.), with the Sr···Sr separation of 4.1548 (8) Å. A 3-D supramolecular network structure is formed through the extended hydrogen-bonding interactions between water molecules and carboxylate O atoms (Table 2).

### Experimental

The title complex was prepared by the addition of strontium nitrate (2.12 g, 10 mmol) to a hot aqueous solution of 2-(3-benzoylphenyl)propanoic acid (3.65 g, 10 mmol); the pH was adjusted to 6 with 0.1*M* sodium hydroxide. The solution was allowed to evaporate at room temperature. Colorless prismatic crystals separated from the filtered solution after several days. CH&N analysis. Calc. for C_32_H_30_SrO_8_: C 60.93, H 4.76%. Found: C 60.96, H 4.83%.

### Refinement

The H atoms were placed in calculated positions [C—H = 0.93 and 0.98 Å and *U*_iso_(H) = 1.2*U*_eq_ (C) for aromatic H atoms and methyne H atoms, C—H = 0.96 Å and *U*_iso_(H) = 1.5*U*_eq_ (C) for methyl H atoms] and were included in the refinement in the riding-model approximation. The H atoms of water molecules were located in difference Fourier maps and refined with the O—H distance restrained to 0.85 (1) Å and *U*_iso_(H) = 1.5*U*_eq_(O).

### Crystal data

**Table:**

[Sr(C_16_H_13_O_3_)_2_(H_2_O)_2_]	*F*(000) = 1296
*M**_r_* = 630.18	*D*_x_ = 1.442 Mg m^−^^3^
Monoclinic, *P*2_1_/*n*	Mo *K*α radiation, λ = 0.71073 Å
Hall symbol: -P 2yn	Cell parameters from 11966 reflections
*a* = 18.665 (4) Å	θ = 3.1–25.0°
*b* = 8.0406 (16) Å	µ = 1.91 mm^−^^1^
*c* = 19.377 (4) Å	*T* = 295 K
β = 93.26 (3)°	Prism, colorless
*V* = 2903.4 (10) Å^3^	0.30 × 0.25 × 0.18 mm
*Z* = 4	

### Data collection

**Table:**

Rigaku R-AXIS RAPID diffractometer	5107 independent reflections
Radiation source: fine-focus sealed tube	2941 reflections with *I* > 2σ(*I*)
graphite	*R*_int_ = 0.087
Detector resolution: 10.000 pixels mm^-1^	θ_max_ = 25.0°, θ_min_ = 3.1°
ω scans	*h* = −22→22
Absorption correction: multi-scan (*ABSCOR*; Higashi, 1995)	*k* = −9→9
*T*_min_ = 0.598, *T*_max_ = 0.725	*l* = −23→19
22141 measured reflections	

### Refinement

**Table:**

Refinement on *F*^2^	Secondary atom site location: difference Fourier map
Least-squares matrix: full	Hydrogen site location: inferred from neighbouring sites
*R*[*F*^2^ > 2σ(*F*^2^)] = 0.050	H atoms treated by a mixture of independent and constrained refinement
*wR*(*F*^2^) = 0.136	*w* = 1/[σ^2^(*F*_o_^2^) + (0.0533*P*)^2^ + 1.5847*P*] where *P* = (*F*_o_^2^ + 2*F*_c_^2^)/3
*S* = 1.08	(Δ/σ)_max_ < 0.001
5107 reflections	Δρ_max_ = 0.69 e Å^−^^3^
385 parameters	Δρ_min_ = −0.96 e Å^−^^3^
7 restraints	Extinction correction: *SHELXL97* (Sheldrick, 2008), Fc^*^=kFc[1+0.001xFc^2^λ^3^/sin(2θ)]^-1/4^
Primary atom site location: structure-invariant direct methods	Extinction coefficient: 0.0013 (4)

### Fractional atomic coordinates and isotropic or equivalent isotropic displacement parameters (Å^2^)

**Table:**

	*x*	*y*	*z*	*U*_iso_*/*U*_eq_	
Sr1	0.48764 (2)	0.75146 (5)	0.52610 (2)	0.04083 (19)	
O1W	0.3724 (2)	0.6001 (5)	0.5485 (3)	0.0869 (15)	
H1W1	0.345 (3)	0.620 (7)	0.581 (3)	0.130*	
H1W2	0.373 (4)	0.496 (2)	0.541 (3)	0.130*	
O1	0.58168 (18)	0.9811 (4)	0.46912 (17)	0.0504 (9)	
O2W	0.5312 (2)	0.8995 (4)	0.6376 (2)	0.0639 (11)	
H2W1	0.563 (2)	0.862 (6)	0.667 (2)	0.096*	
H2W2	0.538 (3)	1.0040 (18)	0.634 (3)	0.096*	
O2	0.61427 (19)	0.7219 (4)	0.4837 (2)	0.0614 (10)	
O3	0.5860 (3)	0.6644 (6)	0.1710 (3)	0.121 (2)	
O4	0.4452 (2)	0.7775 (4)	0.39694 (18)	0.0623 (11)	
O5	0.47219 (19)	0.5182 (4)	0.42097 (18)	0.0556 (10)	
O6	0.2021 (3)	0.3645 (6)	0.4167 (3)	0.1007 (16)	
C1	0.6216 (3)	0.8618 (6)	0.4568 (3)	0.0490 (13)	
C2	0.6843 (4)	0.8805 (7)	0.4118 (4)	0.099 (3)	
H2	0.7251	0.8715	0.4457	0.119*	
C3	0.6965 (5)	1.0412 (7)	0.3813 (4)	0.123 (3)	
H3A	0.7422	1.0413	0.3610	0.184*	
H3B	0.6960	1.1254	0.4164	0.184*	
H3C	0.6593	1.0636	0.3463	0.184*	
C4	0.6951 (4)	0.7317 (8)	0.3669 (4)	0.076 (2)	
C5	0.7489 (4)	0.6203 (8)	0.3800 (4)	0.078 (2)	
H5	0.7810	0.6373	0.4178	0.094*	
C6	0.7570 (4)	0.4815 (7)	0.3384 (4)	0.0743 (19)	
H6	0.7945	0.4080	0.3490	0.089*	
C7	0.7110 (4)	0.4507 (7)	0.2822 (3)	0.0685 (17)	
H7	0.7164	0.3562	0.2553	0.082*	
C8	0.6564 (4)	0.5628 (7)	0.2661 (4)	0.0669 (17)	
C9	0.6493 (4)	0.7039 (7)	0.3090 (4)	0.076 (2)	
H9	0.6129	0.7801	0.2981	0.092*	
C10	0.6085 (4)	0.5418 (8)	0.2023 (4)	0.077 (2)	
C11	0.5888 (3)	0.3725 (8)	0.1769 (4)	0.0702 (17)	
C12	0.5806 (4)	0.3459 (10)	0.1070 (5)	0.095 (2)	
H12	0.5883	0.4329	0.0767	0.114*	
C13	0.5614 (5)	0.1937 (13)	0.0815 (5)	0.114 (3)	
H13	0.5582	0.1760	0.0340	0.137*	
C14	0.5467 (4)	0.0669 (11)	0.1253 (6)	0.110 (3)	
H14	0.5320	−0.0357	0.1075	0.133*	
C15	0.5536 (4)	0.0905 (10)	0.1952 (5)	0.098 (2)	
H15	0.5436	0.0043	0.2251	0.117*	
C16	0.5757 (4)	0.2436 (8)	0.2210 (4)	0.0779 (18)	
H16	0.5818	0.2594	0.2686	0.093*	
C17	0.4514 (3)	0.6286 (6)	0.3793 (3)	0.0474 (13)	
C18	0.4340 (3)	0.5796 (6)	0.3037 (3)	0.0538 (14)	
H18	0.4791	0.5412	0.2855	0.065*	
C19	0.4084 (4)	0.7250 (7)	0.2587 (3)	0.084 (2)	
H19A	0.4005	0.6884	0.2118	0.126*	
H19B	0.4440	0.8112	0.2611	0.126*	
H19C	0.3643	0.7674	0.2750	0.126*	
C20	0.3822 (3)	0.4346 (6)	0.2974 (3)	0.0484 (13)	
C21	0.3833 (3)	0.3290 (7)	0.2406 (3)	0.0553 (14)	
H21	0.4158	0.3494	0.2070	0.066*	
C22	0.3376 (3)	0.1952 (7)	0.2326 (3)	0.0620 (16)	
H22	0.3391	0.1272	0.1939	0.074*	
C23	0.2894 (3)	0.1624 (7)	0.2824 (3)	0.0607 (15)	
H23	0.2602	0.0689	0.2784	0.073*	
C24	0.2845 (3)	0.2682 (6)	0.3384 (3)	0.0547 (14)	
C25	0.3314 (3)	0.4039 (6)	0.3452 (3)	0.0535 (14)	
H25	0.3284	0.4751	0.3827	0.064*	
C26	0.2298 (3)	0.2443 (7)	0.3910 (3)	0.0646 (15)	
C27	0.2095 (3)	0.0740 (7)	0.4110 (3)	0.0587 (15)	
C28	0.2581 (4)	−0.0550 (7)	0.4146 (3)	0.0701 (17)	
H28	0.3045	−0.0382	0.4010	0.084*	
C29	0.2382 (5)	−0.2089 (8)	0.4383 (4)	0.089 (2)	
H29	0.2718	−0.2943	0.4417	0.107*	
C30	0.1708 (6)	−0.2376 (10)	0.4565 (4)	0.104 (3)	
H30	0.1580	−0.3428	0.4715	0.125*	
C31	0.1211 (4)	−0.1121 (11)	0.4530 (4)	0.096 (2)	
H31	0.0746	−0.1325	0.4657	0.115*	
C32	0.1397 (3)	0.0456 (8)	0.4305 (3)	0.0731 (18)	
H32	0.1061	0.1311	0.4286	0.088*	

### Atomic displacement parameters (Å^2^)

**Table:**

	*U*^11^	*U*^22^	*U*^33^	*U*^12^	*U*^13^	*U*^23^
Sr1	0.0499 (3)	0.0273 (3)	0.0458 (3)	0.0009 (2)	0.0062 (2)	0.0017 (2)
O1W	0.069 (3)	0.039 (2)	0.158 (5)	−0.001 (2)	0.052 (3)	0.004 (3)
O1	0.054 (2)	0.0337 (19)	0.064 (2)	0.0082 (17)	0.0082 (18)	0.0007 (17)
O2W	0.095 (3)	0.042 (2)	0.053 (3)	0.003 (2)	−0.017 (2)	0.0068 (18)
O2	0.064 (2)	0.035 (2)	0.089 (3)	0.0039 (17)	0.029 (2)	0.0076 (18)
O3	0.170 (6)	0.065 (3)	0.127 (5)	0.017 (3)	−0.008 (4)	0.032 (3)
O4	0.097 (3)	0.035 (2)	0.053 (2)	0.0004 (19)	−0.015 (2)	0.0016 (16)
O5	0.076 (3)	0.0331 (19)	0.057 (2)	0.0020 (18)	−0.0084 (19)	0.0084 (17)
O6	0.103 (4)	0.059 (3)	0.145 (5)	0.002 (3)	0.047 (3)	−0.016 (3)
C1	0.055 (3)	0.035 (3)	0.058 (4)	−0.004 (3)	0.009 (3)	−0.006 (3)
C2	0.132 (6)	0.045 (4)	0.130 (6)	−0.018 (4)	0.091 (5)	−0.021 (4)
C3	0.195 (9)	0.053 (4)	0.131 (7)	−0.034 (5)	0.100 (6)	−0.019 (4)
C4	0.086 (5)	0.050 (4)	0.096 (5)	−0.023 (4)	0.052 (4)	−0.013 (4)
C5	0.076 (4)	0.060 (4)	0.103 (5)	−0.009 (4)	0.043 (4)	−0.014 (4)
C6	0.071 (4)	0.048 (4)	0.106 (6)	−0.002 (3)	0.030 (4)	−0.011 (4)
C7	0.081 (5)	0.051 (4)	0.076 (5)	0.001 (3)	0.033 (4)	−0.009 (3)
C8	0.077 (4)	0.043 (3)	0.083 (5)	0.001 (3)	0.027 (4)	0.010 (3)
C9	0.085 (5)	0.036 (3)	0.114 (6)	0.008 (3)	0.056 (5)	0.015 (3)
C10	0.090 (5)	0.047 (4)	0.099 (6)	0.011 (4)	0.037 (4)	0.019 (4)
C11	0.072 (4)	0.063 (4)	0.076 (5)	0.009 (3)	0.012 (4)	0.016 (4)
C12	0.095 (6)	0.081 (6)	0.108 (7)	0.006 (4)	0.006 (5)	0.012 (5)
C13	0.121 (7)	0.108 (7)	0.111 (7)	0.015 (6)	−0.008 (6)	−0.002 (6)
C14	0.100 (6)	0.079 (6)	0.149 (9)	0.006 (5)	−0.030 (6)	−0.016 (6)
C15	0.087 (5)	0.070 (5)	0.135 (8)	−0.002 (4)	−0.003 (5)	0.020 (5)
C16	0.082 (4)	0.059 (4)	0.095 (5)	0.005 (4)	0.019 (4)	0.016 (4)
C17	0.054 (3)	0.036 (3)	0.051 (3)	−0.009 (3)	−0.003 (3)	0.002 (3)
C18	0.066 (4)	0.046 (3)	0.048 (3)	−0.010 (3)	−0.006 (3)	0.001 (2)
C19	0.121 (6)	0.066 (4)	0.062 (4)	−0.029 (4)	−0.026 (4)	0.021 (3)
C20	0.058 (3)	0.040 (3)	0.046 (3)	0.003 (3)	−0.006 (3)	0.003 (2)
C21	0.072 (4)	0.048 (3)	0.045 (3)	0.002 (3)	−0.007 (3)	0.003 (3)
C22	0.077 (4)	0.047 (3)	0.060 (4)	0.002 (3)	−0.008 (3)	−0.009 (3)
C23	0.069 (4)	0.043 (3)	0.068 (4)	−0.004 (3)	−0.009 (3)	−0.008 (3)
C24	0.056 (3)	0.047 (3)	0.060 (3)	−0.002 (3)	−0.002 (3)	−0.002 (3)
C25	0.060 (4)	0.041 (3)	0.059 (4)	0.003 (3)	−0.009 (3)	−0.005 (3)
C26	0.065 (4)	0.049 (3)	0.078 (4)	−0.004 (3)	0.000 (3)	−0.009 (3)
C27	0.062 (4)	0.057 (4)	0.056 (4)	−0.004 (3)	−0.003 (3)	−0.006 (3)
C28	0.082 (5)	0.057 (4)	0.070 (4)	0.006 (4)	0.001 (3)	−0.002 (3)
C29	0.134 (7)	0.062 (5)	0.073 (5)	0.009 (5)	0.007 (5)	−0.002 (3)
C30	0.173 (9)	0.061 (5)	0.082 (5)	−0.023 (6)	0.028 (6)	−0.002 (4)
C31	0.106 (6)	0.100 (6)	0.085 (5)	−0.038 (5)	0.026 (5)	−0.014 (5)
C32	0.076 (5)	0.074 (4)	0.070 (4)	−0.008 (4)	0.008 (4)	−0.013 (3)

### Geometric parameters (Å, °)

**Table:**

Sr1—O5^i^	2.496 (3)	C11—C12	1.370 (9)
Sr1—O1^ii^	2.514 (3)	C11—C16	1.375 (8)
Sr1—O1W	2.529 (4)	C12—C13	1.361 (11)
Sr1—O2	2.557 (4)	C12—H12	0.9300
Sr1—O2W	2.559 (4)	C13—C14	1.363 (11)
Sr1—O4	2.590 (4)	C13—H13	0.9300
Sr1—O5	2.772 (3)	C14—C15	1.367 (11)
Sr1—O1	2.816 (3)	C14—H14	0.9300
Sr1—C1	3.037 (5)	C15—C16	1.383 (9)
Sr1—C17	3.050 (5)	C15—H15	0.9300
O1W—H1W1	0.85 (6)	C16—H16	0.9300
O1W—H1W2	0.852 (10)	C17—C18	1.535 (7)
O1—C1	1.246 (5)	C18—C19	1.519 (7)
O1—Sr1^ii^	2.514 (3)	C18—C20	1.515 (7)
O2W—H2W1	0.85 (4)	C18—H18	0.9800
O2W—H2W2	0.852 (10)	C19—H19A	0.9600
O2—C1	1.251 (6)	C19—H19B	0.9600
O3—C10	1.220 (7)	C19—H19C	0.9600
O4—C17	1.252 (6)	C20—C25	1.384 (7)
O5—C17	1.246 (5)	C20—C21	1.392 (7)
O5—Sr1^i^	2.496 (3)	C21—C22	1.377 (7)
O6—C26	1.217 (6)	C21—H21	0.9300
C1—C2	1.505 (7)	C22—C23	1.381 (8)
C2—C3	1.445 (6)	C22—H22	0.9300
C2—C4	1.500 (8)	C23—C24	1.386 (7)
C2—H2	0.9800	C23—H23	0.9300
C3—H3A	0.9600	C24—C25	1.400 (7)
C3—H3B	0.9600	C24—C26	1.494 (8)
C3—H3C	0.9600	C25—H25	0.9300
C4—C5	1.358 (9)	C26—C27	1.479 (8)
C4—C9	1.389 (10)	C27—C28	1.378 (8)
C5—C6	1.389 (8)	C27—C32	1.395 (8)
C5—H5	0.9300	C28—C29	1.378 (8)
C6—C7	1.372 (8)	C28—H28	0.9300
C6—H6	0.9300	C29—C30	1.345 (11)
C7—C8	1.382 (8)	C29—H29	0.9300
C7—H7	0.9300	C30—C31	1.369 (10)
C8—C9	1.417 (8)	C30—H30	0.9300
C8—C10	1.494 (9)	C31—C32	1.391 (9)
C9—H9	0.9300	C31—H31	0.9300
C10—C11	1.487 (9)	C32—H32	0.9300
			
O5^i^—Sr1—O1^ii^	150.60 (11)	H3B—C3—H3C	109.5
O5^i^—Sr1—O1W	75.52 (13)	C5—C4—C9	117.3 (6)
O1^ii^—Sr1—O1W	87.76 (11)	C5—C4—C2	122.7 (8)
O5^i^—Sr1—O2	77.86 (11)	C9—C4—C2	120.0 (7)
O1^ii^—Sr1—O2	125.34 (10)	C4—C5—C6	121.7 (7)
O1W—Sr1—O2	145.14 (12)	C4—C5—H5	119.1
O5^i^—Sr1—O2W	89.06 (11)	C6—C5—H5	119.1
O1^ii^—Sr1—O2W	73.17 (12)	C7—C6—C5	121.4 (6)
O1W—Sr1—O2W	108.15 (16)	C7—C6—H6	119.3
O2—Sr1—O2W	93.41 (14)	C5—C6—H6	119.3
O5^i^—Sr1—O4	122.37 (11)	C6—C7—C8	118.8 (6)
O1^ii^—Sr1—O4	80.61 (11)	C6—C7—H7	120.6
O1W—Sr1—O4	89.33 (16)	C8—C7—H7	120.6
O2—Sr1—O4	86.23 (13)	C7—C8—C9	118.9 (7)
O2W—Sr1—O4	147.50 (11)	C7—C8—C10	120.7 (6)
O5^i^—Sr1—O5	74.36 (12)	C9—C8—C10	120.3 (6)
O1^ii^—Sr1—O5	125.00 (11)	C4—C9—C8	121.9 (7)
O1W—Sr1—O5	75.31 (14)	C4—C9—H9	119.1
O2—Sr1—O5	76.14 (12)	C8—C9—H9	119.1
O2W—Sr1—O5	161.83 (11)	O3—C10—C11	120.2 (7)
O4—Sr1—O5	48.03 (10)	O3—C10—C8	119.5 (6)
O5^i^—Sr1—O1	123.47 (11)	C11—C10—C8	120.3 (6)
O1^ii^—Sr1—O1	77.71 (11)	C12—C11—C16	118.9 (7)
O1W—Sr1—O1	159.64 (14)	C12—C11—C10	118.8 (6)
O2—Sr1—O1	47.69 (10)	C16—C11—C10	122.2 (7)
O2W—Sr1—O1	81.43 (12)	C13—C12—C11	120.7 (8)
O4—Sr1—O1	74.41 (11)	C13—C12—H12	119.6
O5—Sr1—O1	101.34 (10)	C11—C12—H12	119.6
O5^i^—Sr1—C1	101.54 (13)	C12—C13—C14	120.3 (9)
O1^ii^—Sr1—C1	101.82 (13)	C12—C13—H13	119.8
O1W—Sr1—C1	160.99 (16)	C14—C13—H13	119.8
O2—Sr1—C1	23.92 (11)	C13—C14—C15	120.1 (9)
O2W—Sr1—C1	90.44 (14)	C13—C14—H14	119.9
O4—Sr1—C1	76.29 (14)	C15—C14—H14	119.9
O5—Sr1—C1	85.78 (12)	C14—C15—C16	119.5 (8)
O1—Sr1—C1	24.20 (10)	C14—C15—H15	120.3
O5^i^—Sr1—C17	98.46 (13)	C16—C15—H15	120.3
O1^ii^—Sr1—C17	102.95 (13)	C11—C16—C15	120.4 (8)
O1W—Sr1—C17	81.86 (16)	C11—C16—H16	119.8
O2—Sr1—C17	80.23 (13)	C15—C16—H16	119.8
O2W—Sr1—C17	168.83 (13)	O5—C17—O4	122.3 (5)
O4—Sr1—C17	23.91 (11)	O5—C17—C18	118.5 (5)
O5—Sr1—C17	24.12 (11)	O4—C17—C18	119.2 (4)
O1—Sr1—C17	87.53 (12)	O5—C17—Sr1	65.3 (3)
C1—Sr1—C17	80.01 (13)	O4—C17—Sr1	57.0 (3)
O5^i^—Sr1—Sr1^ii^	154.28 (8)	C18—C17—Sr1	175.9 (3)
O1^ii^—Sr1—Sr1^ii^	41.47 (7)	C19—C18—C20	111.6 (4)
O1W—Sr1—Sr1^ii^	127.72 (10)	C19—C18—C17	113.1 (5)
O2—Sr1—Sr1^ii^	83.90 (7)	C20—C18—C17	111.9 (4)
O2W—Sr1—Sr1^ii^	73.97 (8)	C19—C18—H18	106.6
O4—Sr1—Sr1^ii^	73.69 (7)	C20—C18—H18	106.6
O5—Sr1—Sr1^ii^	118.70 (7)	C17—C18—H18	106.6
O1—Sr1—Sr1^ii^	36.24 (7)	C18—C19—H19A	109.5
C1—Sr1—Sr1^ii^	60.37 (10)	C18—C19—H19B	109.5
C17—Sr1—Sr1^ii^	96.11 (10)	H19A—C19—H19B	109.5
O5^i^—Sr1—Sr1^i^	39.46 (8)	C18—C19—H19C	109.5
O1^ii^—Sr1—Sr1^i^	153.66 (8)	H19A—C19—H19C	109.5
O1W—Sr1—Sr1^i^	71.59 (9)	H19B—C19—H19C	109.5
O2—Sr1—Sr1^i^	73.55 (7)	C25—C20—C21	117.4 (5)
O2W—Sr1—Sr1^i^	128.12 (8)	C25—C20—C18	122.9 (5)
O4—Sr1—Sr1^i^	82.92 (7)	C21—C20—C18	119.7 (5)
O5—Sr1—Sr1^i^	34.90 (7)	C22—C21—C20	122.0 (5)
O1—Sr1—Sr1^i^	117.22 (7)	C22—C21—H21	119.0
C1—Sr1—Sr1^i^	94.03 (10)	C20—C21—H21	119.0
C17—Sr1—Sr1^i^	59.01 (10)	C21—C22—C23	119.6 (5)
Sr1^ii^—Sr1—Sr1^i^	148.45 (2)	C21—C22—H22	120.2
Sr1—O1W—H1W1	126 (5)	C23—C22—H22	120.2
Sr1—O1W—H1W2	115 (4)	C22—C23—C24	120.4 (5)
H1W1—O1W—H1W2	109 (6)	C22—C23—H23	119.8
C1—O1—Sr1^ii^	168.6 (3)	C24—C23—H23	119.8
C1—O1—Sr1	87.9 (3)	C23—C24—C25	118.8 (5)
Sr1^ii^—O1—Sr1	102.29 (11)	C23—C24—C26	122.2 (5)
Sr1—O2W—H2W1	125 (4)	C25—C24—C26	118.9 (5)
Sr1—O2W—H2W2	115 (4)	C20—C25—C24	121.7 (5)
H2W1—O2W—H2W2	108 (5)	C20—C25—H25	119.1
C1—O2—Sr1	100.1 (3)	C24—C25—H25	119.1
C17—O4—Sr1	99.1 (3)	O6—C26—C27	120.4 (6)
C17—O5—Sr1^i^	163.7 (3)	O6—C26—C24	120.0 (5)
C17—O5—Sr1	90.5 (3)	C27—C26—C24	119.6 (5)
Sr1^i^—O5—Sr1	105.64 (12)	C28—C27—C32	119.0 (6)
O1—C1—O2	122.1 (5)	C28—C27—C26	122.1 (6)
O1—C1—C2	121.8 (5)	C32—C27—C26	118.7 (6)
O2—C1—C2	116.0 (5)	C29—C28—C27	120.2 (7)
O1—C1—Sr1	67.9 (3)	C29—C28—H28	119.9
O2—C1—Sr1	56.0 (3)	C27—C28—H28	119.9
C2—C1—Sr1	166.3 (4)	C30—C29—C28	121.0 (8)
C3—C2—C4	116.5 (6)	C30—C29—H29	119.5
C3—C2—C1	118.2 (5)	C28—C29—H29	119.5
C4—C2—C1	113.0 (5)	C29—C30—C31	120.1 (7)
C3—C2—H2	101.8	C29—C30—H30	120.0
C4—C2—H2	101.8	C31—C30—H30	120.0
C1—C2—H2	101.8	C30—C31—C32	120.5 (7)
C2—C3—H3A	109.5	C30—C31—H31	119.8
C2—C3—H3B	109.5	C32—C31—H31	119.8
H3A—C3—H3B	109.5	C31—C32—C27	119.2 (6)
C2—C3—H3C	109.5	C31—C32—H32	120.4
H3A—C3—H3C	109.5	C27—C32—H32	120.4
			
O5^i^—Sr1—O1—C1	27.6 (3)	O1—C1—C2—C3	−1.5 (10)
O1^ii^—Sr1—O1—C1	−174.7 (3)	O2—C1—C2—C3	−178.0 (7)
O1W—Sr1—O1—C1	−129.3 (4)	Sr1—C1—C2—C3	131.0 (16)
O2—Sr1—O1—C1	8.1 (3)	O1—C1—C2—C4	−142.6 (6)
O2W—Sr1—O1—C1	110.8 (3)	O2—C1—C2—C4	41.0 (9)
O4—Sr1—O1—C1	−91.2 (3)	Sr1—C1—C2—C4	−10 (2)
O5—Sr1—O1—C1	−51.0 (3)	C3—C2—C4—C5	112.9 (9)
C17—Sr1—O1—C1	−70.9 (3)	C1—C2—C4—C5	−105.4 (7)
Sr1^ii^—Sr1—O1—C1	−174.7 (3)	C3—C2—C4—C9	−66.8 (9)
Sr1^i^—Sr1—O1—C1	−17.9 (3)	C1—C2—C4—C9	74.9 (8)
O5^i^—Sr1—O1—Sr1^ii^	−157.66 (11)	C9—C4—C5—C6	−1.6 (8)
O1^ii^—Sr1—O1—Sr1^ii^	0.0	C2—C4—C5—C6	178.7 (5)
O1W—Sr1—O1—Sr1^ii^	45.4 (4)	C4—C5—C6—C7	0.0 (9)
O2—Sr1—O1—Sr1^ii^	−177.2 (2)	C5—C6—C7—C8	1.4 (9)
O2W—Sr1—O1—Sr1^ii^	−74.52 (13)	C6—C7—C8—C9	−1.0 (8)
O4—Sr1—O1—Sr1^ii^	83.54 (13)	C6—C7—C8—C10	175.2 (5)
O5—Sr1—O1—Sr1^ii^	123.71 (12)	C5—C4—C9—C8	1.9 (9)
C1—Sr1—O1—Sr1^ii^	174.7 (3)	C2—C4—C9—C8	−178.4 (5)
C17—Sr1—O1—Sr1^ii^	103.83 (14)	C7—C8—C9—C4	−0.6 (8)
Sr1^i^—Sr1—O1—Sr1^ii^	156.83 (7)	C10—C8—C9—C4	−176.9 (5)
O5^i^—Sr1—O2—C1	−171.7 (3)	C7—C8—C10—O3	−145.4 (6)
O1^ii^—Sr1—O2—C1	−11.6 (4)	C9—C8—C10—O3	30.8 (9)
O1W—Sr1—O2—C1	147.5 (4)	C7—C8—C10—C11	34.2 (8)
O2W—Sr1—O2—C1	−83.4 (3)	C9—C8—C10—C11	−149.6 (6)
O4—Sr1—O2—C1	64.1 (3)	O3—C10—C11—C12	34.6 (10)
O5—Sr1—O2—C1	111.7 (3)	C8—C10—C11—C12	−145.0 (6)
O1—Sr1—O2—C1	−8.2 (3)	O3—C10—C11—C16	−142.9 (7)
C17—Sr1—O2—C1	87.4 (3)	C8—C10—C11—C16	37.5 (9)
Sr1^ii^—Sr1—O2—C1	−9.9 (3)	C16—C11—C12—C13	−1.3 (11)
Sr1^i^—Sr1—O2—C1	147.8 (3)	C10—C11—C12—C13	−178.9 (7)
O5^i^—Sr1—O4—C17	1.2 (4)	C11—C12—C13—C14	3.0 (13)
O1^ii^—Sr1—O4—C17	−159.0 (3)	C12—C13—C14—C15	−2.2 (13)
O1W—Sr1—O4—C17	−71.1 (3)	C13—C14—C15—C16	−0.1 (13)
O2—Sr1—O4—C17	74.3 (3)	C12—C11—C16—C15	−1.1 (10)
O2W—Sr1—O4—C17	164.7 (3)	C10—C11—C16—C15	176.4 (6)
O5—Sr1—O4—C17	−0.4 (3)	C14—C15—C16—C11	1.8 (11)
O1—Sr1—O4—C17	121.3 (3)	Sr1^i^—O5—C17—O4	−174.3 (9)
C1—Sr1—O4—C17	96.3 (3)	Sr1—O5—C17—O4	−0.8 (5)
Sr1^ii^—Sr1—O4—C17	159.0 (3)	Sr1^i^—O5—C17—C18	4.7 (15)
Sr1^i^—Sr1—O4—C17	0.4 (3)	Sr1—O5—C17—C18	178.2 (4)
O5^i^—Sr1—O5—C17	−178.1 (4)	Sr1^i^—O5—C17—Sr1	−173.5 (13)
O1^ii^—Sr1—O5—C17	26.6 (3)	Sr1—O4—C17—O5	0.9 (6)
O1W—Sr1—O5—C17	103.1 (3)	Sr1—O4—C17—C18	−178.2 (4)
O2—Sr1—O5—C17	−97.1 (3)	O5^i^—Sr1—C17—O5	1.8 (4)
O2W—Sr1—O5—C17	−153.3 (4)	O1^ii^—Sr1—C17—O5	−157.9 (3)
O4—Sr1—O5—C17	0.4 (3)	O1W—Sr1—C17—O5	−72.1 (3)
O1—Sr1—O5—C17	−56.3 (3)	O2—Sr1—C17—O5	77.9 (3)
C1—Sr1—O5—C17	−74.9 (3)	O2W—Sr1—C17—O5	133.8 (6)
Sr1^ii^—Sr1—O5—C17	−22.2 (3)	O4—Sr1—C17—O5	−179.2 (5)
Sr1^i^—Sr1—O5—C17	−178.1 (4)	O1—Sr1—C17—O5	125.3 (3)
O5^i^—Sr1—O5—Sr1^i^	0.0	C1—Sr1—C17—O5	102.2 (3)
O1^ii^—Sr1—O5—Sr1^i^	−155.29 (11)	Sr1^ii^—Sr1—C17—O5	160.6 (3)
O1W—Sr1—O5—Sr1^i^	−78.75 (16)	Sr1^i^—Sr1—C17—O5	1.3 (2)
O2—Sr1—O5—Sr1^i^	81.03 (14)	O5^i^—Sr1—C17—O4	−179.0 (3)
O2W—Sr1—O5—Sr1^i^	24.8 (4)	O1^ii^—Sr1—C17—O4	21.3 (3)
O4—Sr1—O5—Sr1^i^	178.6 (2)	O1W—Sr1—C17—O4	107.1 (3)
O1—Sr1—O5—Sr1^i^	121.86 (12)	O2—Sr1—C17—O4	−103.0 (3)
C1—Sr1—O5—Sr1^i^	103.23 (15)	O2W—Sr1—C17—O4	−47.1 (9)
C17—Sr1—O5—Sr1^i^	178.1 (4)	O5—Sr1—C17—O4	179.2 (5)
Sr1^ii^—Sr1—O5—Sr1^i^	155.97 (8)	O1—Sr1—C17—O4	−55.5 (3)
Sr1^ii^—O1—C1—O2	−167.6 (13)	C1—Sr1—C17—O4	−78.7 (3)
Sr1—O1—C1—O2	−14.6 (5)	Sr1^ii^—Sr1—C17—O4	−20.2 (3)
Sr1^ii^—O1—C1—C2	16 (2)	Sr1^i^—Sr1—C17—O4	−179.6 (4)
Sr1—O1—C1—C2	169.1 (6)	O5—C17—C18—C19	179.2 (5)
Sr1^ii^—O1—C1—Sr1	−153.0 (17)	O4—C17—C18—C19	−1.7 (7)
Sr1—O2—C1—O1	16.4 (6)	O5—C17—C18—C20	52.1 (7)
Sr1—O2—C1—C2	−167.2 (5)	O4—C17—C18—C20	−128.8 (5)
O5^i^—Sr1—C1—O1	−156.7 (3)	C19—C18—C20—C25	−98.5 (6)
O1^ii^—Sr1—C1—O1	5.3 (3)	C17—C18—C20—C25	29.4 (7)
O1W—Sr1—C1—O1	124.3 (4)	C19—C18—C20—C21	79.5 (7)
O2—Sr1—C1—O1	−165.1 (5)	C17—C18—C20—C21	−152.6 (5)
O2W—Sr1—C1—O1	−67.6 (3)	C25—C20—C21—C22	−2.2 (8)
O4—Sr1—C1—O1	82.4 (3)	C18—C20—C21—C22	179.8 (5)
O5—Sr1—C1—O1	130.2 (3)	C20—C21—C22—C23	−0.7 (8)
C17—Sr1—C1—O1	106.6 (3)	C21—C22—C23—C24	3.3 (9)
Sr1^ii^—Sr1—C1—O1	3.6 (2)	C22—C23—C24—C25	−3.0 (8)
Sr1^i^—Sr1—C1—O1	164.1 (3)	C22—C23—C24—C26	175.8 (5)
O5^i^—Sr1—C1—O2	8.3 (3)	C21—C20—C25—C24	2.4 (8)
O1^ii^—Sr1—C1—O2	170.3 (3)	C18—C20—C25—C24	−179.6 (5)
O1W—Sr1—C1—O2	−70.7 (6)	C23—C24—C25—C20	0.1 (8)
O2W—Sr1—C1—O2	97.4 (3)	C26—C24—C25—C20	−178.7 (5)
O4—Sr1—C1—O2	−112.5 (3)	C23—C24—C26—O6	−143.7 (6)
O5—Sr1—C1—O2	−64.8 (3)	C25—C24—C26—O6	35.1 (8)
O1—Sr1—C1—O2	165.1 (5)	C23—C24—C26—C27	35.8 (8)
C17—Sr1—C1—O2	−88.4 (3)	C25—C24—C26—C27	−145.4 (5)
Sr1^ii^—Sr1—C1—O2	168.6 (4)	O6—C26—C27—C28	−145.7 (6)
Sr1^i^—Sr1—C1—O2	−30.8 (3)	C24—C26—C27—C28	34.8 (8)
O5^i^—Sr1—C1—C2	65.8 (19)	O6—C26—C27—C32	30.7 (9)
O1^ii^—Sr1—C1—C2	−132.2 (19)	C24—C26—C27—C32	−148.8 (5)
O1W—Sr1—C1—C2	−13 (2)	C32—C27—C28—C29	−1.1 (9)
O2—Sr1—C1—C2	57.5 (18)	C26—C27—C28—C29	175.3 (6)
O2W—Sr1—C1—C2	154.9 (19)	C27—C28—C29—C30	1.8 (10)
O4—Sr1—C1—C2	−55.1 (19)	C28—C29—C30—C31	−1.3 (12)
O5—Sr1—C1—C2	−7.3 (19)	C29—C30—C31—C32	0.1 (12)
O1—Sr1—C1—C2	−137 (2)	C30—C31—C32—C27	0.6 (10)
C17—Sr1—C1—C2	−30.9 (19)	C28—C27—C32—C31	−0.1 (9)
Sr1^ii^—Sr1—C1—C2	−133.9 (19)	C26—C27—C32—C31	−176.6 (6)
Sr1^i^—Sr1—C1—C2	26.6 (19)		

Symmetry codes: (i) −*x*+1, −*y*+1, −*z*+1; (ii) −*x*+1, −*y*+2, −*z*+1.

### Hydrogen-bond geometry (Å, °)

**Table:**

*D*—H···*A*	*D*—H	H···*A*	*D*···*A*	*D*—H···*A*
O1W—H1W2···O2^i^	0.85 (1)	1.83 (1)	2.678 (5)	171 (6)
O2W—H2W2···O4^ii^	0.85 (1)	1.89 (2)	2.724 (5)	166 (6)

Symmetry codes: (i) −*x*+1, −*y*+1, −*z*+1; (ii) −*x*+1, −*y*+2, −*z*+1.

## References

[bb1] Higashi, T. (1995). *ABSCOR* Rigaku Corporation, Tokyo, Japan.

[bb2] Johnson, C. K. (1976). *ORTEPII* Report ORNL-5138. Oak Ridge National Laboratory, Tennessee, USA.

[bb3] Rigaku (1998). *RAPID-AUTO* Rigaku Corporation, Tokyo, Japan.

[bb4] Rigaku/MSC (2002). *CrystalStructure* Rigaku/MSC, The Woodlands, Texas, USA.

[bb5] Sheldrick, G. M. (2008). *Acta Cryst.* A**64**, 112–122. 10.1107/S0108767307043930 18156677

[bb6] Tahir, M. N., Ülkü, D., Ali, S., Masood, T., Danish, M. & Mazhar, M. (1997). *Acta Cryst.* C**53**, 1574–1576.

[bb7] Zhang, Z.-Y., Chen, P.-G., Deng, Z.-P., Yu, N. & Liu, B.-Y. (2007*a*). *Acta Cryst.* E**63**, m1900–m1901.

[bb8] Zhang, Z.-Y., Yu, N., Guo, X.-X., Pu, J. & Sun, J.-P. (2007*b*). *Acta Cryst.* E**63**, m2883.

